# Passivation of Lithium Metal Anode via Hybrid Ionic Liquid Electrolyte toward Stable Li Plating/Stripping

**DOI:** 10.1002/advs.201600400

**Published:** 2016-11-03

**Authors:** Nian‐Wu Li, Ya‐Xia Yin, Jin‐Yi Li, Chang‐Huan Zhang, Yu‐Guo Guo

**Affiliations:** ^1^ CAS Key Laboratory of Molecular Nanostructure and NanotechnologyInstitute of ChemistryChinese Academy of Sciences (CAS)Beijing100190P. R. China; ^2^ School of Chemistry and Chemical EngineeringUniversity of Chinese Academy of SciencesBeijing100049P. R. China

**Keywords:** electrochemistry, hybrid electrolyte, ionic liquid, lithium metal, lithium metal batteries

## Abstract

**Hybrid electrolyte of ionic liquid and ethers** is used to passivate the surface of Li metal surface via modification of the as‐formed solid electrolyte interphase with *N*‐propyl‐*N*‐methylpyrrolidinium bis(trifluoromethanesulfonyl)amide (Py_13_TFSI), thereby reducing the side reactions between the Li metal and electrolyte, leading to remarkably suppressed Li dendrite growth and mitigating Li metal corrosion.

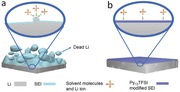

High energy electrochemical storage systems are urgently needed to satisfy the continuously surging demand in consumer electronics, electric vehicles, and grid storage. Unfortunately, the energy density of existing battery systems remains insufficient for many of the aforementioned applications.^1^ Li metal is a promising anode material for energy storage systems because Li metal has an extremely high theoretical specific capacity (3860 mA h g^−1^) and the lowest negative electrochemical potential (−3.04 V vs the standard hydrogen electrode) among all elements.^2^ However, the commercial of rechargeable Li metal batteries has been hindered by the low Coulombic efficiency of Li deposition/dissolution, uncontrolled Li dendrite growth, and accumulation of “dead Li” or corrosion of Li metal anode.^3^ Li metal reacts spontaneously with most organic electrolyte solvents and Li salts to instantly form a solid electrolyte interphase (SEI) layer on the Li metal surface because Li is thermodynamically unstable in organic electrolytes.^4^ The SEI layer is ionically conductive but electronically insulating and thus can prevent further reaction between Li metal and electrolyte. However, the brittle SEI layer cannot withstand the mechanical deformation resulted from the Li plating/stripping processes. Thus, the SEI layer continuously break and repair during cycling, and the Li dendrite are grown at the site where the current density is locally enhanced.[[qv: 4b]] Due to the growth of the Li dendrite, the surface area of the Li surface increase markedly, thereby increasing the side reaction between Li metal and electrolyte and decreasing the Coulombic efficiency of Li plating/stripping processes. Eventually, some of the Li dendrite may become electrically isolated or “dead Li” because of the uneven plating/stripping of the Li dendrites. The repeated breakage and repair of the SEI layer consumes both Li metal and electrolyte, leading to the drying up of electrolyte and the severe corrosion of Li metal anode, which is the key factor of the battery degradation and failure.^3^

There are numerous reports which attempt to solve these problems. Various strategies have been designed to prevent dendrite penetration and reduce dendrite structure, including the use of nanostructured anodes,^5^ modified separators,^6^ and physical protective layers.^7^ However, these strategies cannot change the breakage/repair mechanism of SEI layer, and significantly improve the Coulombic efficiency of Li plating/stripping. In addition, optimization of electrolyte using additives,^8^ high concentrated electrolytes,^9^ and ionic liquid electrolytes^10^ can enhance the stability of SEI layers, and can improve the Coulombic efficiency of Li plating/stripping. However, the electrolyte additives are consumed during the SEI formation and thus affect the electrochemical performance. Both the high concentrated electrolytes and ionic liquid electrolytes have high viscosity and low conductivity, which are adverse to the practical Li metal battery performance. Most recently, passivation of Li metal anode is considered as a promising way to enhance the SEI layers and reduce the dendrite growth.^11^ However, the degeneration of passivation film is irreversible because the passivation film cannot regenerate in the electrolyte during cycling.

Herein, we demonstrate a promising Li metal passivation strategy by using hybrid *N*‐propyl‐*N*‐methylpyrrolidinium bis(trifluoromethanesulfonyl)amide (Py_13_TFSI) and ether electrolyte. The reversibility of Li plating/stripping can be remarkably increased by the synergy between Py_13_TFSI ionic liquid and Li salt concentration. The hybrid electrolyte can enhance the stability of SEI layer by the in situ passivation process. The Li dendrite growth and the corrosion of Li metal anode can be efficiently restrained during cycling of Li metal batteries (**Figure** **1**).

**Figure 1:**
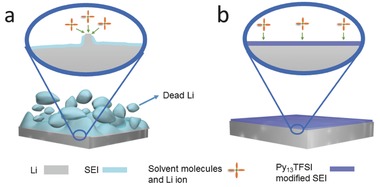
Schematic diagrams of Li metal structures in different electrolytes in the Li metal batteries. a) Ether based electrolyte and b) optimized hybrid electrolyte.

The Li|Cu cells were used to evaluate the Coulombic efficiency and cycling stability of different electrolytes. With the increase of the concentration of Li salt from 1 to 2 m, both Py_13_TFSI ionic liquid and Li salt concentration in the electrolyte are helpful to the improvement of the Coulombic efficiency of Li plating/stripping (**Figure** **2** and Figure S1 (Supporting Information)). However, the Coulombic efficiency of Li plating/stripping is not obviously increased with the increase of Li salt concentration from 2 to 4 m (Figure S1, Supporting Information). Thus, the synergy between Py_13_TFSI ionic liquid and Li salt concentration plays an important role in improving the Coulombic efficiency of Li plating/stripping. Remarkably, the voltage of Li plating/stripping curves increases with increasing Li salt concentration from 2 to 4 m. With the increase of Li salt concentration (2–4 m), the viscosity of the Py_13_TFSI based electrolytes increases significantly (Figure S2, Supporting Information), and the ionic conductivity obviously decreases (Figure S3, Supporting Information), respectively. The high viscosity and low conductivity are adverse to the reversibility of Li plating/stripping.^12^ Thus, the 2 m Li salt concentration in the hybrid electrolyte is the optimal choose for Li metal batteries. The Li|Cu cell using the optimized hybrid electrolyte shows high cycling stability, and the Coulombic efficiency of Li plating/stripping is 99.1% after 360 cycles (Figure 2c), which is close to or better than the previously reports.[[qv: 5a,8c]] Furthermore, the Li|Cu cell using the optimized hybrid electrolyte also shows high Coulombic efficiency of Li plating/stripping at high practical areal capacities of 3.0 mA h cm^−2^ with current density of 1 mA cm^−2^ (Figure S4, Supporting Information).

**Figure 2:**
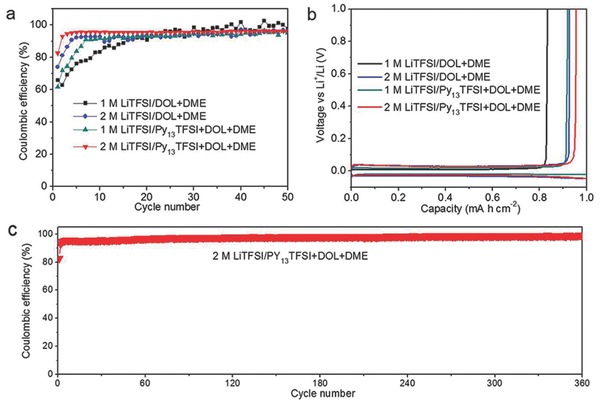
a) The Coulombic efficiency of Li plating/stripping using various electrolytes and b) corresponding voltage profiles in Li|Cu cells. c) The Coulombic efficiency of Li plating/stripping using hybrid electrolytes during long cycling in Li|Cu cells.

To further investigate the morphology of Li metal anode in the practical Li metal batteries using different electrolytes, commercial LiFePO_4_ cathodes were used as the counter electrodes. After ten cycles in Li|LiFePO_4_ batteries, Li metal anode using dimethoxyethane (DME) and dioxolane (DOL) based electrolyte exhibits a moss‐like dendrite structure (**Figure** **3**a). In comparison, the Li metal anode using optimized hybrid electrolyte displays a smooth surface without dendrite structure (Figure 3b). The smooth surface of Li metal anode can reduce the side reactions between the deposition Li and electrolyte, thereby leading to higher reversibility of Li plating/stripping. Seen from the energy‐dispersive X‐ray spectroscopy mapping images (Figure S5, Supporting Information), the C, O, N, S, and F are uniformly distributed on the surface of the Li metal anode. In addition, the Li|LiFePO_4_ batteries with various current density were used to evaluate the performance of the optimized hybrid electrolyte in restraining the Li dendrite growth (Figure 3c–j). The Li metal anode exhibits smooth surface, even in the current density of 0.5 mA cm^−2^. Although the Li metal anode exhibits porous structure at the current density of 1 mA cm^−2^, the dendrite structure can be effectively reduced.

**Figure 3:**
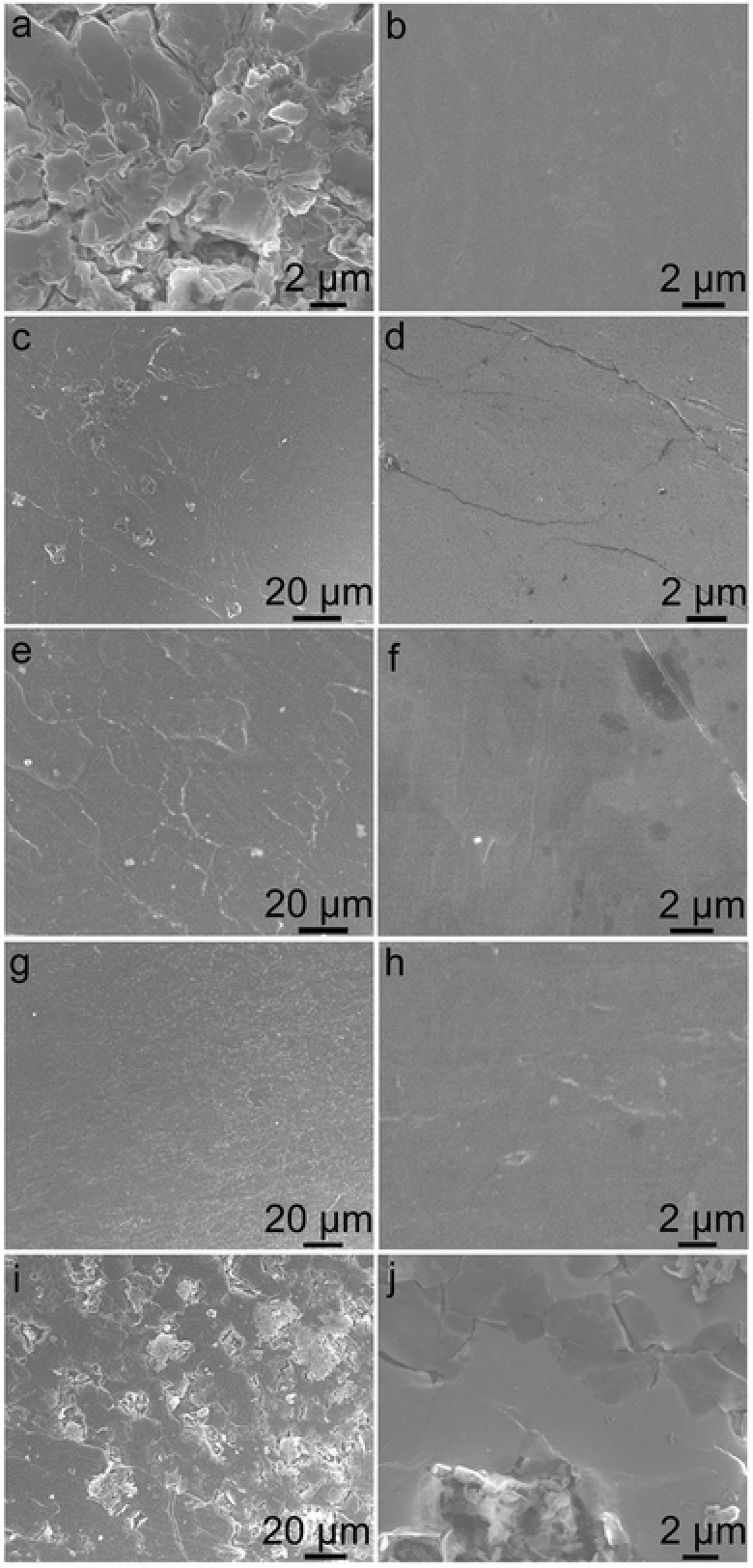
Scanning electron microscope (SEM) images of Li metal anode. The morphology of Li metal anode in the Li|LiFePO_4_ batteries after ten cycles using a) 2 m LiTFSI/DOL‐DME electrolyte and b) 2 m LiTFSI/Py_13_TFSI‐DOL‐DME electrolyte, and c) 2 m LiTFSI/Py_13_TFSI‐DOL‐DME electrolyte at current density of c,d) 0.1 mA cm^−2^, e,f) 0.2 mA cm^−2^, g,h) 0.5 mA cm^−2^, and i,j) 1 mA cm^−2^, respectively.

The X‐ray photoelectron spectroscopy (XPS) was employed to analyze the element and valence of the SEI layer during cycling in the Li|LiFePO_4_ batteries (**Figure** **4**a–h and Table S1 (Supporting Information)). As shown in the C 1s spectra, the decomposition products of solvents including CO_3_^2−^, COOR, COR, and C—C are founded on the surface of the Li metal after cycling, which agree with the previous reports.^13^ The DOL decomposition product (COOR) increases after ten cycles in the ether based electrolyte. The major difference lies in the spectra of N 1s and F 1s. In the spectra of N 1s, the peak around 399.7 eV is assigned to the imide groups in the LiTFSI salt or Py_13_TFSI.[[qv: 13a]] Two extra peaks around 397.2 and 402.6 eV are attributed to the N^−^ in Li_3_N, and the N^+^ in Py_13_^+^, respectively.^14^ There is no obvious change in the concentrations of the Py_13_^+^ and TFSI^−^ on the surface of the Li metal anode after ten cycles in the optimized hybrid electrolyte. While, the concentration of Li_3_N increase obviously after ten cycles. Thus, in the optimized hybrid electrolyte system, the SEI layers on Li metal surface are modified by the Py_13_TFSI ionic liquid, thereby forming a passivation interphase. The passivation interphase is stable during cycling and the side reactions between electrolyte and Li metal is reduced. This electrolyte system also promotes the generation of Li_3_N during cycling, which is advantageous for preventing the Li dendrite growth.^15^

**Figure 4:**
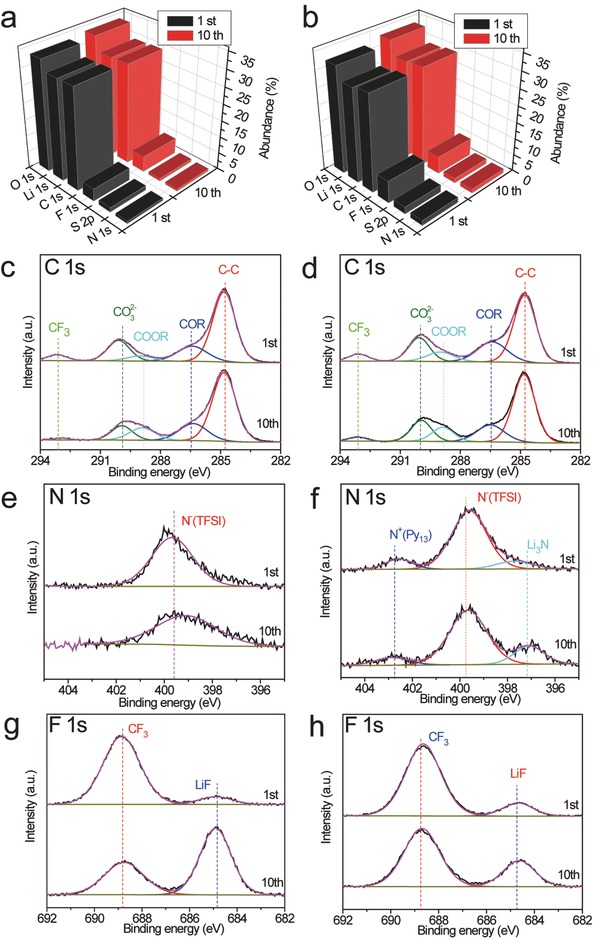
XPS results of Li metal anode using different electrolytes. The atomic composition of Li metal and corresponding XPS spectra using a,c,e,g) 2 m LiTFSI/DOL‐DME electrolyte and b,d,f,h) 2 m LiTFSI/Py_13_TFSI‐DOL‐DME electrolyte, respectively.

In the spectra of F 1s, the concentration of LiF on the surface of Li metal anode increases markedly, and the —CF_3_ functional group decreases sharply after ten cycles in the Li|LiFePO_4_ battery using ether based electrolyte. In contrast, the concentration of LiF increases slightly, and the —CF_3_ functional group decreases slightly after ten cycles in the Li|LiFePO_4_ battery using optimized hybrid electrolyte. Recently, Archer and co‐workers[[qv: 8a]] reported that addition of LiF to the conventional electrolyte could effectively restrain the growth of Li dendrites. In our experiment, LiF is only the decomposition product of LiTFSI on the surface of the Li metal, and the concentration of the LiF is limited in the SEI layers. Therefore, it could not play an important role in controlling the Li dendrites growth. Seen from the S 2p spectra (Figure S6, Supporting Information), the Li*_x_*SO*_y_* species increase after ten cycles in the Li metal battery using ether based electrolytes. In comparison, there is no obvious change in the concentration of the Li*_x_*SO*_y_* species on the surface of Li metal anode in the optimized hybrid electrolyte. Thus, the optimized hybrid electrolyte system has inhibiting effect on the decomposition of the LiTFSI and generation of LiF and Li*_x_*SO*_y_*.

Interfacial stability and charge transfer behavior were investigated by symmetrical cell testing (**Figure** **5**a). The results show that the voltage hysteresis keeps increasing with cycling in the ether based electrolyte because of Li dendrite growth and gradually accumulated SEI layer, which agree with the previous reports.[[qv: 5b]] While, the cell displays a much lower voltage fluctuation in the optimized hybrid electrolyte, indicating a uniform Li deposition with a stable and thin SEI layer, causing excellent charge transfer kinetics. Additionally, the results of the electrochemical impedance spectroscopy also show that the cell using optimized hybrid electrolyte exhibits higher interfacial stability (Figure S7, Supporting Information).

**Figure 5:**
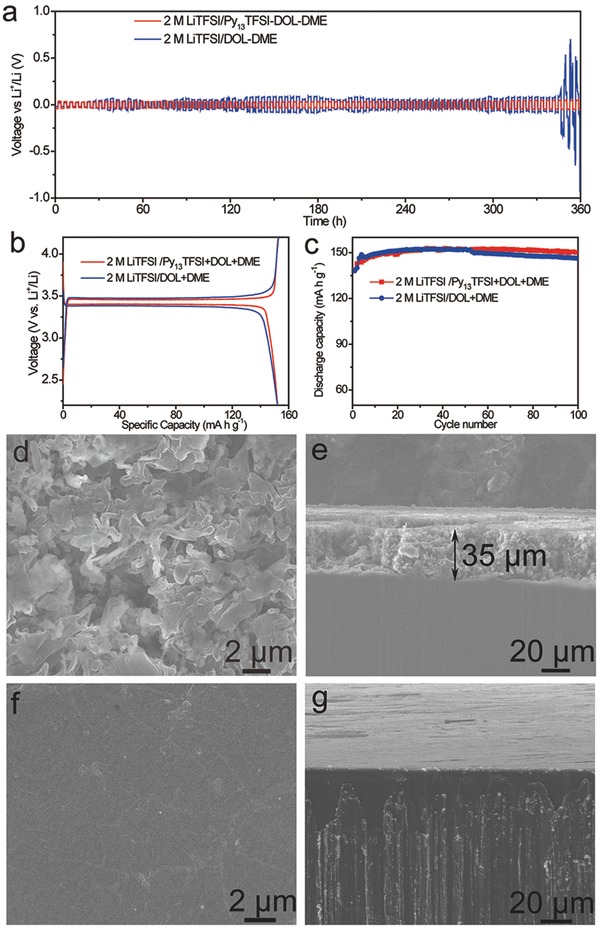
Cycling performance of Li|Li symmetric cell and Li|LiFePO_4_ battery. a) The comparison of voltage profiles of Li plating/stripping in Li|Li symmetric cells. b) Cycling performance and c) corresponding discharge/charge curves of Li|LiFePO_4_ batteries using different electrolytes. d) The top‐view SEM image and e) side‐view SEM image of Li metal anode after 100 cycles in Li|LiFePO_4_ battery using ether based electrolyte. f) The top‐view SEM image and g) side‐view SEM image of Li metal anode after 100 cycles in Li|LiFePO_4_ battery using optimized hybrid electrolyte.

The performance of the Li|LiFePO_4_ batteries using different electrolytes is shown in the Figure 5. The capacity increase is attributed to the activation process of LiFePO_4_ materials.[[qv: 11a,16]] The better cycling stability of the cell using the optimized hybrid electrolyte is attributed to its stable interface in the Li|LiFePO_4_ battery system. In comparison with the carbonate electrolyte in the previous reports, the ether based electrolyte in this paper can reduce the corrosion of Li metal anode slightly because of the high Li salt concentration and their lower reactivity with Li metal.[[qv: 9a,11a]] However, after 100 cycles, the Li metal anode using ether based electrolyte also exhibits dendrite structure and severe corrosion of Li metal anode (Figure 5d,e). While, the Li metal anode using optimized hybrid electrolyte exhibits smooth surface, and the corrosion of Li metal anode is restrained (Figure 5f,g).

According to the analysis of the results, the main composition of SEI layers in the Li|LiFePO_4_ batteries using ether based electrolyte is Li_2_CO_3_, Li*_x_*SO*_y_*, LiCOOR, COR, C—C, and LiF. Some of the side reaction products increased markedly with continuous cycling of Li metal batteries. This phenomenon is attributed to the continuous breakage/repair mechanism of SEI layers and the continuous Li dendrite growth.[[qv: 4b,15a]] The continuous Li dendrite growth leads to a porous structure with high surface area, thereby accelerating the irreversibly reaction between Li metal and electrolyte.[[qv: 2c]] Eventually, some of the Li dendrite may become electrically isolated or “dead Li” (Figure 1) because of the uneven plating/stripping of the Li dendrites.[[qv: 13a]] Thus, the corrosion of Li metal anode is very serious, and amount of “dead Li” can be seen from the cross‐section image of the Li metal anode (Figure 5d,e). While, in the optimized hybrid electrolyte system, the SEI layers on the Li metal surface is modified by Py_13_TFSI ionic liquid, thereby forming a passivation interphase. The side reaction products do not obviously increase during the cycling. Thus, the passivation interphase can efficiently reduce the side reactions between Li metal and electrolyte, and restrain the uneven plating/stripping of the Li metal. Therefore, the Li metal anode shows a compact and smooth surface after 100 cycles in the Li metal batteries, and the Li dendrite growth and the corrosion of Li metal anode have been restrained (Figure 5f,g). Additionally, the optimized hybrid electrolyte can significantly improve Coulombic efficiency of Li plating/stripping because of the synergy between Py_13_TFSI ionic liquid and Li salt concentration.

In conclusion, we demonstrated that the Li dendrite growth and the corrosion of Li metal anode issues in the Li metal batteries can be effectively restrained by the surface passivation in the optimized hybrid ionic liquid electrolyte. The synergy between Py_13_TFSI ionic liquid and Li salt concentration can remarkably improve the reversibility of Li plating/stripping. The stability of SEI layers on Li metal can be remarkably enhanced by Py_13_TFSI ionic liquid modifying in the passivation process. Our work illustrates a passivation strategy for solving the Li dendrite growth and corrosion of Li metal anode issues in advanced energy storage systems.

## Supporting information

As a service to our authors and readers, this journal provides supporting information supplied by the authors. Such materials are peer reviewed and may be re‐organized for online delivery, but are not copy‐edited or typeset. Technical support issues arising from supporting information (other than missing files) should be addressed to the authors.

Supplementary
