# Supplementary material for: Passivation of Lithium Metal Anode via Hybrid Ionic Liquid Electrolyte toward Stable Li Plating/Stripping
Source: Adv Sci (Weinh). 2016 Nov 3;4(2):1600400. doi: 10.1002/advs.201600400 (PMC5323882; doi:10.1002/advs.201600400)
Supplement: Supplementary file 1 — Supplementary [file ADVS-4-na-s001.pdf]

Copyright WILEY-VCH Verlag GmbH & Co. KGaA, 69469 Weinheim, Germany, 2016.

ADVANCED SCIENCE Open Access

Supporting Information

for *Adv. Sci.,* DOI: 10.1002/advs.201600400

Passivation of Lithium Metal Anode via Hybrid Ionic Liquid Electrolyte toward Stable Li Plating/Stripping

*Nian-Wu Li, Ya-Xia Yin, Jin-Yi Li, Chang-Huan Zhang, and Yu-Guo Guo**

Copyright WILEY-VCH Verlag GmbH & Co. KGaA, 69469 Weinheim, Germany, 2016.

Supporting Information for

Passivation of Lithium Metal Anode via Hybrid Ionic Liquid Electrolyte towards Stable Li Plating/Stripping

*Nian-Wu Li, Ya-Xia Yin, Jin-Yi Li, Chang-Huan Zhang, and Yu-Guo Guo**

Dr. N.-W. Li, Dr. Y.-X. Yin, J.-Y. Li, Dr. C.-H. Zhang, Prof. Y.-G. Guo
CAS Key Laboratory of Molecular Nanostructure and Nanotechnology, Institute of Chemistry, Chinese Academy of Sciences (CAS), Beijing 100190 (P. R. China)
E-mail: ygguo@iccas.ac.cn
J.-Y. Li, Dr. Y.-X. Yin, Prof. Y.-G. Guo
School of Chemistry and Chemical Engineering, University of Chinese Academy of Sciences, Beijing 100049 (P. R. China)

Experimental Section

Materials. The lithium bis(trifluoromethanesulfonyl)imide (LiTFSI), 1,2-dimethoxyethane (DME), 1,3-dioxolane (DOL) were purchased from Sigma Aldrich. The *N*-propyl-*N*-methylpyrrolidinium bis(trifluoromethanesulfonyl)amide (Py_{13}TFSI) ionic liquid was purchased from Lanzhou kaite trade Co., Ltd.

Electrochemical testing. The cathode slurry was prepared by mixing 80 wt% $LiFePO_4$, 10 wt% Super P, and 10 wt% polyvinylidene difluoride (PVDF, Alfa Aesar) dissolved in *N*-methyl-2-pyrrolidone (NMP, Aldrich). The areal loading of the $LiFePO_4$ cathode is about 4 mg cm^{-2}. The cathodes were produced by coating the slurry onto aluminum foil and drying at 80 °C for 12 h. The cell tests were performed using coin cells cycled at room temperature between 2.2 V and 4.2 V; the cells were fabricated in an argon-filled glove box using polished Li metal as the counter electrode and Celgard 2325 as the separator. The performance of the cells was tested using a LAND electrochemical testing system.

Symmetric Li|Li coin cells were prepared using two Li metal foils, a separator, and electrolyte. For the voltage profile, the current density is 0.5 mA cm^{-2}, and the amount of Li plated in each cycle is 1 mA h cm^{-2}. The EIS measurements were performed on a Princeton PARSTAT MC 1000 multi-channel electrochemical workstation over the frequency range from 100 mHz to 100 kHz with an amplitude of 10 mV.

Materials characterization. To gain insight into the Li plating/stripping in the Li metal battery, we disassembled the coin cells in a glove box. The anodes were washed with DOL-DME (1:1, by volume) three times to remove the residual electrolyte. After washing, the Li electrodes were dried under vacuum for 1 h to remove the residual DOL-DME. The anodes were transferred to some special designed devices for *ex situ* scanning electron microscope (SEM) and X-ray photoelectron spectroscopy (XPS) characterization without exposing the samples to air. The microstructure of the samples was examined with a JEOL 6710F field-emission scanning electron microscope (FE-SEM). XPS was performed on a Thermo Scientific ESCALab 250Xi using 200 W monochromated Al-Kα radiation. The viscosity of each electrolyte was determined by a NiRun SNB-1 viscometer at 25 °C. The conductivity of each electrolyte was measured using a REX DDS-307 conductivity meter at 25 °C.

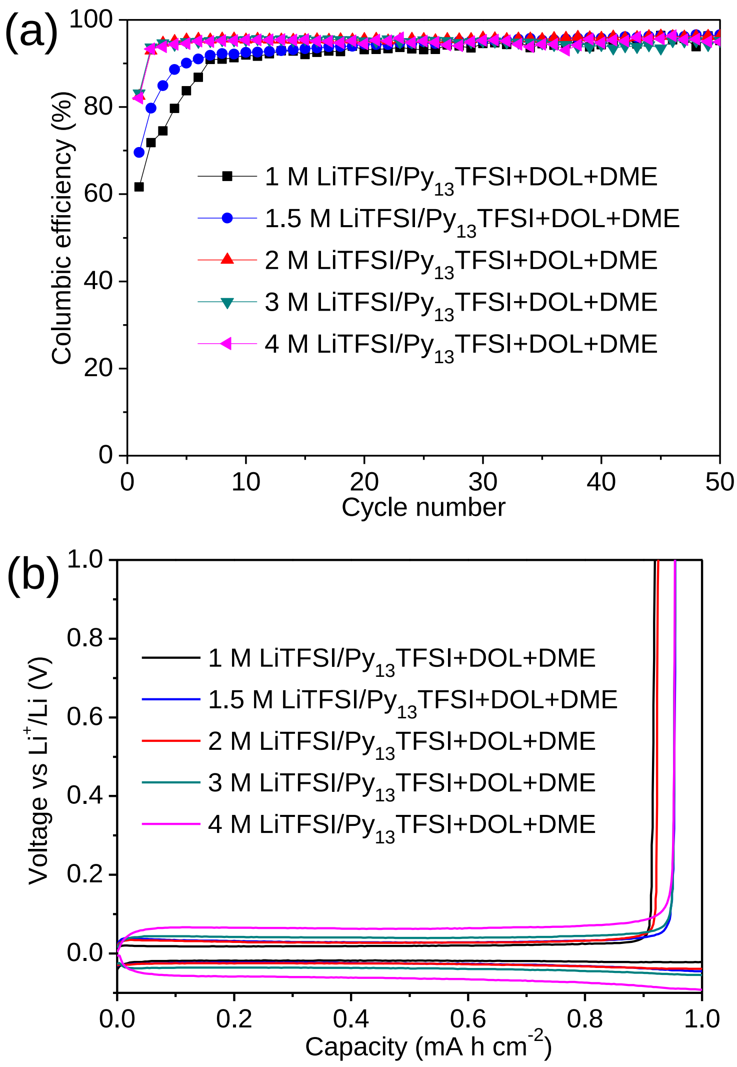

Figure S1. The Coulombic efficiency of Li plating/stripping (a) and corresponding voltage profiles (b) using hybrid electrolyte with different Li salt concentration.

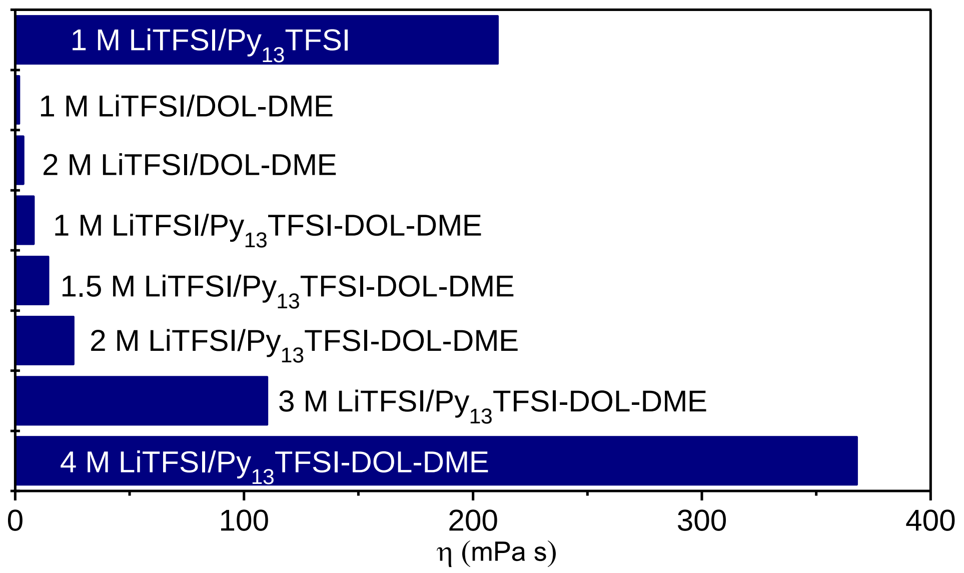

Figure S2. The viscosity of different electrolytes.

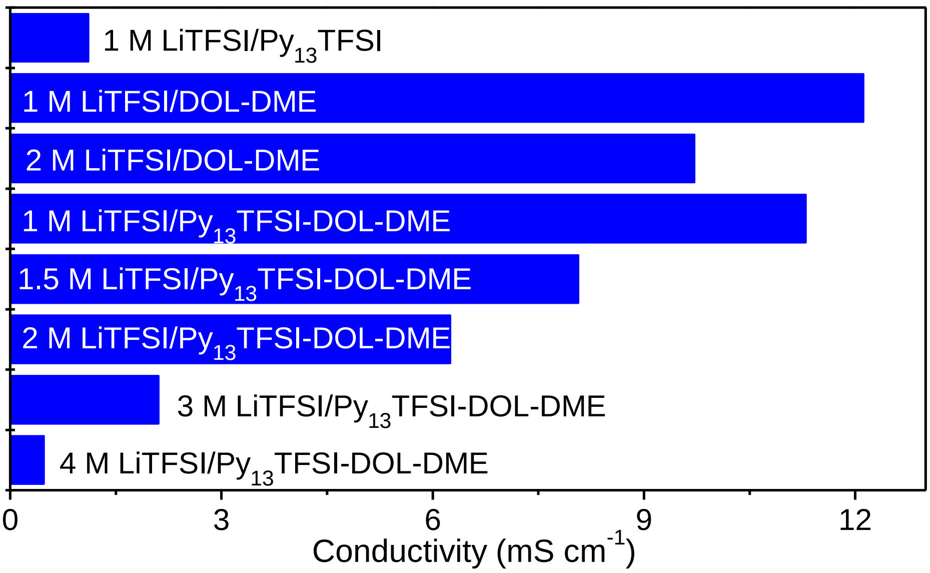

Figure S3. The conductivity of different electrolytes.

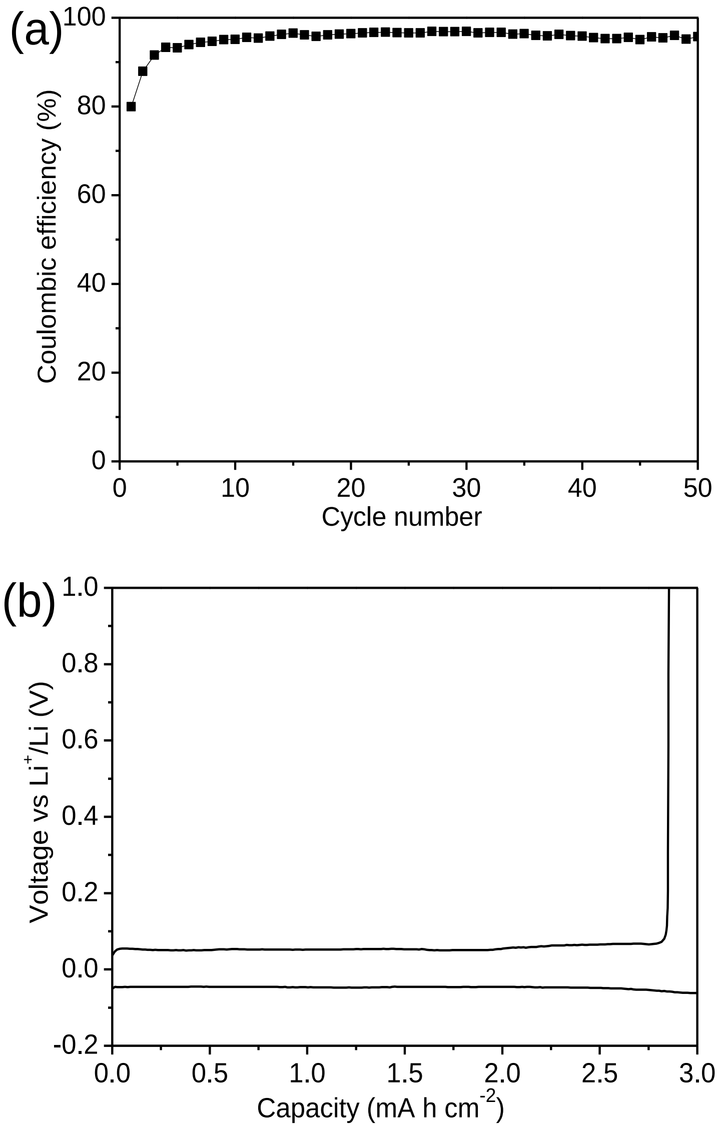

Figure S4. The Coulombic efficiency of Li plating/stripping (a) and corresponding voltage profiles (b) using the optimized hybrid electrolyte at current density of 1 mA cm^{-2}.

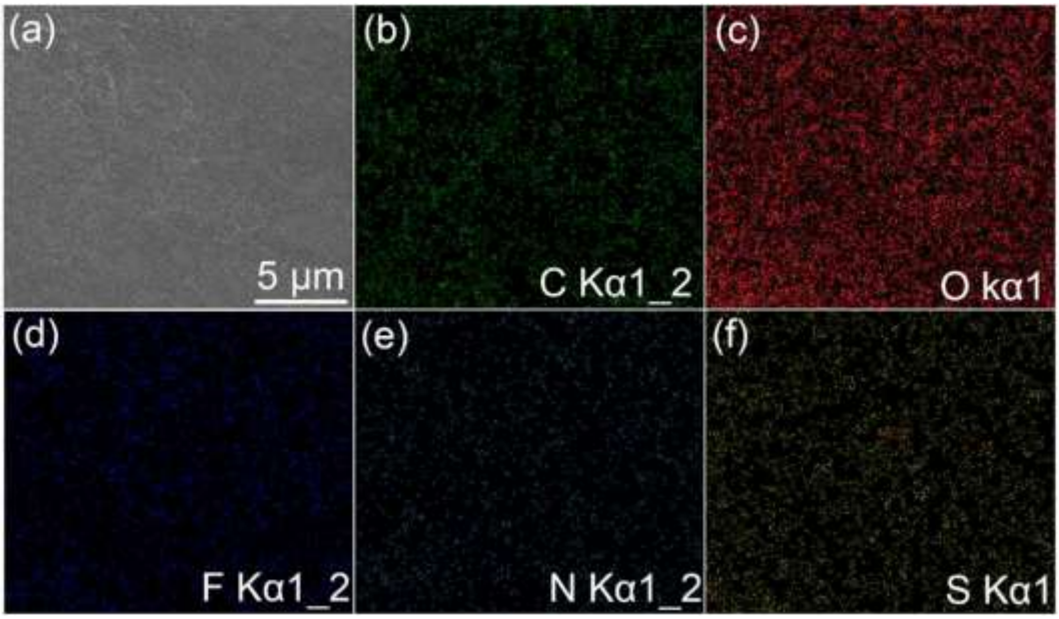

Figure S5. SEM images of Li metal anode and corresponding element mapping after 10 cycles in Li|$LiFePO_4$ battery using optimized hybrid electrolyte.

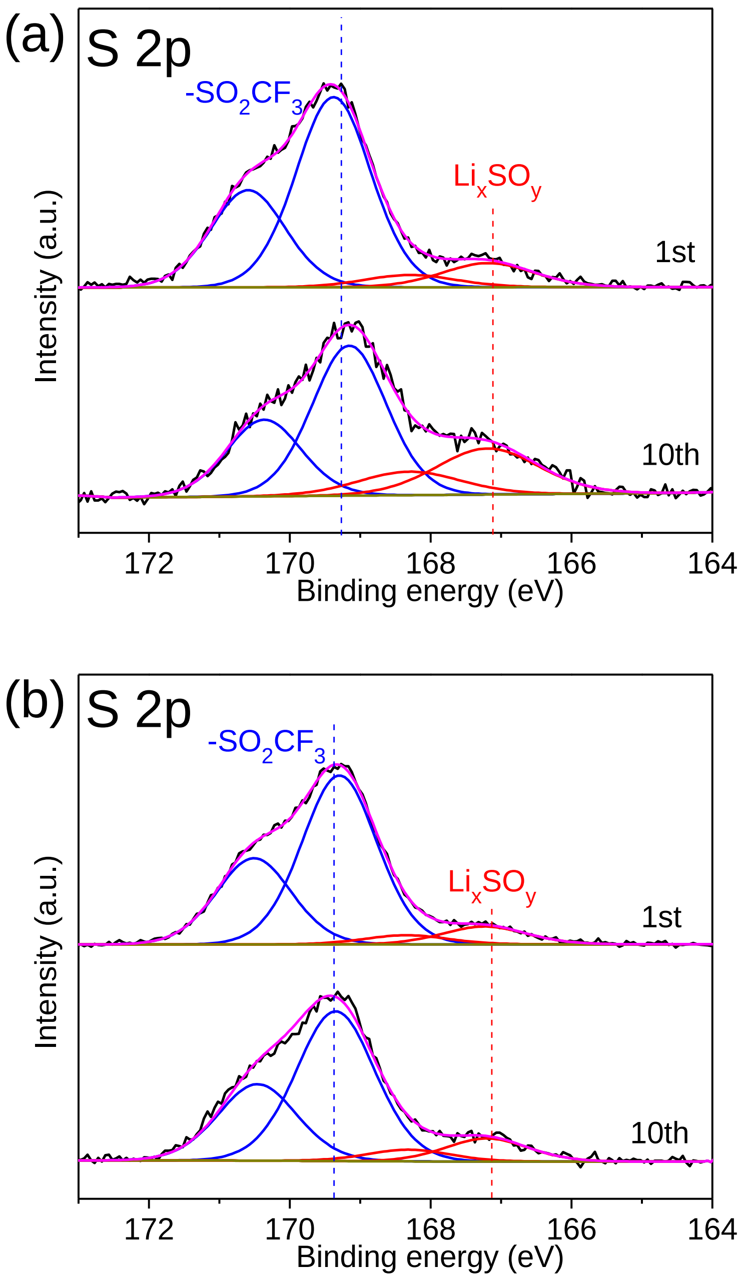

Figure S6. The XPS spectra of S 2p for Li metal anode using ether based electrolyte (a) and optimized hybrid electrolyte (b).

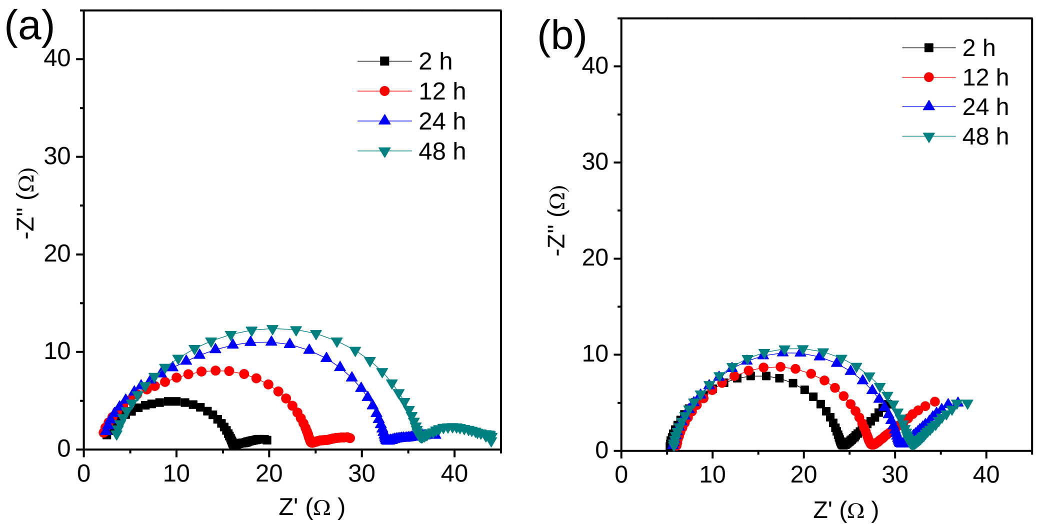

Figure S7. Nyquist plots of Li|Li symmetric cell using ether based electrolyte (a) and optimized hybrid electrolyte, respectively.

The electrochemical impedance spectroscopy (EIS) measurements were performed on the symmetric cell. In the Nyquist plots (Figure S7), the semicircle at high frequency is associated to the resistance through the SEI layer (R_{SEI}). The R_{SEI} value continuously increases with time, which is attributed to the reaction between the Li metal and the organic electrolytes. In comparison of the cell using ether based electrolyte, the cell using optimized hybrid electrolyte exhibits high interfacial stability.

Table S1. XPS binding energies with attributed species and their assumed origin.

Spectra details	Binding energy (eV)	Attributed species	reference	Assumed origin
C 1s	293.1	$-CF_3$	[1]	$TFSI^-$ in LiTFSI or $Py_{13}TFSI$
	290.0	CO_3^{2-}	[1-2]	Carbonates (solvent decomposition products)
	288.9	COOR	[1b, 3]	Carbonyl or polymeric species (DOL decomposition products)
	286.4	COR	[1b, 2-3]	DOL or DME decomposition products
	284.8	C-C	[1-2]	DOL or DME decomposition products
N 1s	397.2	Li_3N	[1a, 4]	LiTFSI decomposition products
	399.7	N^-(TFSI)	[1a, 3-4]	$TFSI^-$ in LiTFSI or $Py_{13}TFSI$
	402.6	$N^+(Py_{13})$	[1a, 4]	Py_{13}^+ in $Py_{13}TFSI$
F 1s	684.8	LiF	[1a, 5]	LiTFSI decomposition products
	688.7	$-CF_3$	[1a, 5]	$TFSI^{-1}$
S 2p	169.3	$-SO_2CF_3$	[3-4]	$TFSI^{-1}$ or $TFSI^{-1}$ decomposition products
	167.2	Li_xSO_y	[3-4]	LiTFSI decomposition products

References

[1] a) P. C. Howlett, N. Brack, A. F. Hollenkamp, M. Forsyth, D. R. MacFarlane, *J Electrochem Soc* **2006**, *153*, A595-A606; b) W. Li, H. Yao, K. Yan, G. Zheng, Z. Liang, Y.-M. Chiang, Y. Cui, *Nat Commun* **2015**, *6*.

[2] S. Z. Xiong, K. Xie, Y. Diao, X. B. Hong, *J Power Sources* **2014**, *246*, 840-845.

[3] M. R. Busche, T. Drossel, T. Leichtweiss, D. A. Weber, M. Falk, M. Schneider, M. L. Reich, H. Sommer, P. Adelhelm, J. Janek, *Nat Chem* **2016**, *8*, 426-434.

[4] J. M. Zheng, M. Gu, H. H. Chen, P. Meduri, M. H. Engelhard, J. G. Zhang, J. Liu, J. Xiao, *J Mater Chem A* **2013**, *1*, 8464-8470.

[5] P. C. Howlett, D. R. MacFarlane, A. F. Hollenkamp, *Electrochem Solid St* **2004**, *7*, A97-A101.
